# The Conserved Tarp Actin Binding Domain Is Important for Chlamydial Invasion

**DOI:** 10.1371/journal.ppat.1000997

**Published:** 2010-07-15

**Authors:** Travis J. Jewett, Natalie J. Miller, Cheryl A. Dooley, Ted Hackstadt

**Affiliations:** Host-Parasite Interactions Section, Laboratory of Intracellular Parasites, Rocky Mountain Laboratories, National Institute of Allergy and Infectious Diseases, National Institutes of Health, Hamilton, Montana, United States of America; University of California San Francisco, United States of America

## Abstract

The translocated actin recruiting phosphoprotein (Tarp) is conserved among all pathogenic chlamydial species. Previous reports identified single *C. trachomatis* Tarp actin binding and proline rich domains required for Tarp mediated actin nucleation. A peptide antiserum specific for the Tarp actin binding domain was generated and inhibited actin polymerization *in vitro* and *C. trachomatis* entry *in vivo*, indicating an essential role for Tarp in chlamydial pathogenesis. Sequence analysis of Tarp orthologs from additional chlamydial species and *C. trachomatis* serovars indicated multiple putative actin binding sites. In order to determine whether the identified actin binding domains are functionally conserved, GST-Tarp fusions from multiple chlamydial species were examined for their ability to bind and nucleate actin. Chlamydial Tarps harbored variable numbers of actin binding sites and promoted actin nucleation as determined by *in vitro* polymerization assays. Our findings indicate that Tarp mediated actin binding and nucleation is a conserved feature among diverse chlamydial species and this function plays a critical role in bacterial invasion of host cells.

## Author Summary

Chlamydiae are bacterial obligate intracellular pathogens responsible for multiple human and veterinary diseases. The induction of cytoskeletal rearrangements to promote chlamydial internalization is partially mediated by a type III secreted effector protein called Tarp that is translocated upon contact with host cells and independently nucleates actin filament formation. Tarp from a *C. trachomatis* lymphogranuloma venereum (LGV) strain consists of a tyrosine-rich repeat domain, a proline-rich domain required for oligomerization, and a single actin binding domain. Oligomerization is required to bring multiple actin monomers together to initiate actin filament formation by a mechanism distinct from host actin nucleators. Here we have examined Tarp from several other strains of chlamydiae and find that certain of these contain up to four actin binding domains. Tarp fragments bearing multiple actin binding domains nucleate actin in *in vitro* assays even in the absence of the oligomerization domain. This suggests that Tarp from different chlamydial species may utilize hybrid mechanisms to induce actin nucleation. Determination of virulence determinants in chlamydiae is challenging due to the lack of tractable genetic systems. The direct introduction of anti-Tarp actin binding domain antibodies into the cytosol of host cells inhibited entry and thus demonstrates an essential role for Tarp in chlamydial pathogenesis.

## Introduction

The obligate intracellular gram negative bacterium, *Chlamydia trachomatis*, is the most frequently reported sexually transmitted disease in the United States and the leading cause of preventable blindness worldwide [1]. This genetically intractable microorganism undergoes a developmental cycle that involves an extracellular infectious form referred to as an elementary body (EB) and an intracellular replicative reticulate body (RB). EB to RB and RB to EB transitions occur within the protective confines of a membrane bound parasitophorous vacuole termed an inclusion [2].

The exact mechanisms of chlamydial attachment and entry of nonphagocytic cells are unclear, but certain features, such as the recruitment of actin to the site of attachment, are conserved among all chlamydial species examined thus far [3]. Furthermore, drugs such as cytochalasin D that prevent actin polymerization inhibit infection [3]–[7]. Like many intracellular microorganisms, chlamydial pathogenesis is partially mediated by the translocation of secreted effectors at the time of bacterium-host-cell contact [8]–[13]. Recent studies have shown that *C. trachomatis* directly induces actin polymerization via the type III secreted effector, Tarp [14]. *C. trachomatis* elementary bodies harbor presynthesized Tarp effector protein which is tyrosine phosphorylated upon translocation to the cytosol of the host cell and has been implicated in the recruitment of actin observed at the site of chlamydial attachment *in vivo* [9]. Tarp was subsequently shown to polymerize actin independently of host factors *in vitro* [14]. The mechanism of Tarp actin nucleation appears to be distinct from known eukaryotic actin nucleators [15]. *Chlamydia trachomatis* L2 Tarp harbors at least three functionally distinct domains; an N-terminal tyrosine-rich repeat domain of unknown function, a proline rich domain required for Tarp oligomerization and a single Wiskott-Aldrich syndrome protein (WASP)-Homology-2 (WH2) G-actin binding domain [9], [14]. The proline rich domain and actin binding domain are harbored within the minimum Tarp peptide required for Tarp mediated actin nucleation. Oligomerization mediated by the proline-rich domain presumably brings multiple actin monomers into apposition to nucleate actin filament formation [14]. Sequence analysis of all known Tarp orthologs indicate that the proline rich domain and actin binding alpha helix are conserved although the tyrosine-rich repeat domain is absent from *C. caviae*, *C. muridarum*, and *C. pneumoniae* [16].

Overall, Tarp orthologs show a low level of sequence identity [16], however the actin binding domains appears to be a conserved feature. We demonstrate here that Tarp orthologs from *Chlamydia pneumonia*, *C. caviae*, *C. muridarum* and various *C. trachomatis* serovars all harbor at least one and up to four functional actin binding domains and that purified recombinant Tarps from all chlamydial species were capable of nucleating actin filament formation *in vitro*. Furthermore, a peptide antiserum with affinity to the actin binding domain prevented Tarp mediated actin nucleation and, when delivered into host cells, significantly reduced host cell susceptibility to chlamydial infection. Our findings show that Tarp mediated actin polymerization is a conserved feature among diverse chlamydial species and suggest that the actin binding domain plays an important role in bacterial entry.

## Results

### A peptide antibody recognizes the actin binding domain of *C. trachomatis* L2 Tarp

**Figure 1:**
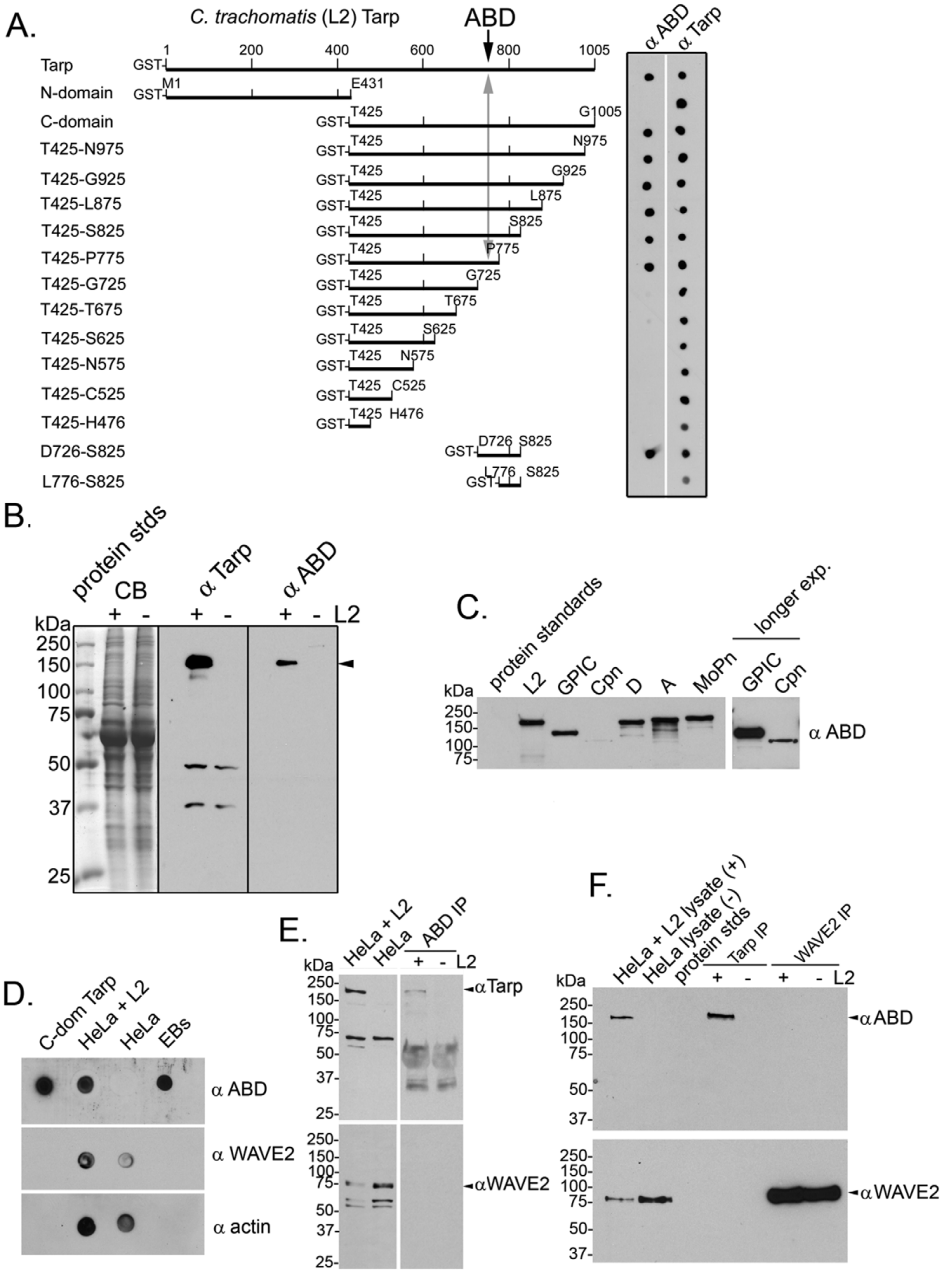
The Tarp actin binding domain (ABD) peptide antibody recognizes native Tarp of multiple serovars and species and does not recognize the ABD of the host cell WAVE2 protein. **A**) Schematic of *C. trachomatis* GST-Tarp fusions used to examine the specificity of the peptide antibody directed toward the Tarp actin binding domain. Tarp amino acids and positions are indicated above each bar in the schematic. Purified GST fusions were immobilized to nitrocellulose and immunoblots were performed with Tarp actin binding domain (αABD) and Tarp (α Tarp) specific antibodies. **B**) The Tarp actin binding domain (α ABD) specific antisera recognizes only a single protein within chlamydia-infected host cells. Chlamydia-infected (+L2) and uninfected (−L2) host cells were suspended in protein sample buffer following a 30 min. infection. Proteins were resolved by SDS-PAGE and visualized by Coomassie blue staining (CB). Immunoblots were performed with Tarp (α Tarp) and Tarp actin binding domain (α ABD) specific antisera. **C**) The Tarp actin binding domain (α ABD) antibody recognizes a protein in lysates generated from purified *C. trachomatis* serovar L2 (LGV-434), *C. caviae* (GPIC), *C. pneumoniae* (Cpn), *C. trachomatis* serovar D (D-UW3), *C. trachomatis* serovar A (A HAR-13) and *C. muridarum* mouse pneumonitis biovar (MoPn) elementary bodies. Loading for SDS-PAGE was based upon equivalent numbers of EBs. *C. pneumoniae* Tarp was not readily visible on the original exposure but was easily visualized with longer exposures. **D**) The Tarp actin binding domain (α ABD) antibody recognizes non-reduced, non-denatured native protein immobilized to nitrocellulose. Immunoblots were performed of lysates generated from cells infected with *C. trachomatis* (HeLa +L2) and uninfected host cells (HeLa). Purified recombinant Tarp protein (C-domain Tarp) and solubilized lysates derived from elementary bodies (EBs) served as positive controls. Immunoblots to detect WAVE2 (α WAVE2) and actin (α actin) were performed as additional controls. **E**) The Tarp actin binding domain (α ABD) antibody immunoprecipitates Tarp from infected cells. Tarp was immunoprecipitated with α ABD from lysates generated from cells infected with *C. trachomatis* (HeLa +L2) and uninfected host cells (HeLa). Proteins were resolved by SDS-PAGE and immunoblotted with Tarp (α Tarp) and WAVE2 (α WAVE2) specific antibodies (arrowheads). The anti-Tarp polyclonal antibody recognizes an unknown antigen in the infected and uninfected HeLa cell lysates that is not immunoprecipitated by the α ABD antibody. The αABD antibody does not recognize this antigen in immunoblots (panels B, D, and F). Note that the IgG heavy chain is observed in both infected and uninfected lanes. **F**) Tarp and WAVE2 were immunoprecipitated from infected (+L2) and uninfected (−L2) HeLa cells, resolved by SDS-PAGE and immunoblotted with Tarp actin binding domain (α ABD) and WAVE2 (α WAVE2) specific antibodies (arrowheads). Molecular mass is in kilodaltons (kDa) for panels B, C, E & F.

Three distinct domains of the *C. trachomatis* Tarp effector have been described. These include: the tyrosine rich repeat region (amino acids 125–424), a proline rich domain required for Tarp multimerization (amino acids 625–650), and an actin binding domain (amino acids 748–758) [9], [14], [16], [17]. Only the proline rich domain and actin binding domain appear to be conserved among chlamydial species. To determine the significance of the actin binding domain, an anti-peptide antibody to amino acids 746–760 encompassing that region was produced in rabbits and antigen-affinity purified to generate the anti actin binding domain (ABD) antibody. This antibody demonstrated affinity to the actin binding domain as confirmed by immunoblots using a series of GST-L2-Tarp fusion proteins (Figure 1A). Western blot analysis of chlamydia-infected host cells with the ABD antibody confirmed the antibody recognizes Tarp in the infected lysates but did not detectably cross-react with any host proteins (Figure 1B). The affinity purified L2 Tarp ABD antibody also recognized an immunodominant protein in lysates generated from purified EBs of other chlamydial species including *C. pneumoniae*, *C. muridarum*, and *C. caviae* (Figure 1C).

Although the ABD antibody was produced against a 15 amino acid peptide antigen, specificity was further examined under more native conditions. *C. trachomatis* L2-infected and uninfected HeLa cells were solubilized in RipA buffer and dot-immunoblotted against the ABD antibody (Figure 1D). This antibody did not detectably cross-react with any host proteins in the whole cell lysate. To confirm this specificity, Tarp was immunoprecipitated from these lysates and probed for WAVE2, which among eukaryotic WH2-domain containing proteins, exhibits the closest similarity to the WH2 domain of Tarp [17], [28] (Figure 1E and F). Again, no cross-reactivity with WAVE2 was observed. Despite our inability to detect reactivity of the ABD antibody to WAVE2 by immunoblotting, immunoprecipitation, or dot blot analysis, we cannot rule out the possibility of low affinity antibody binding to WH2 domains below detectable limits.

### The actin binding domain antibody inhibits actin nucleation *in vitro* and chlamydial invasion *in vivo*

**Figure 2:**
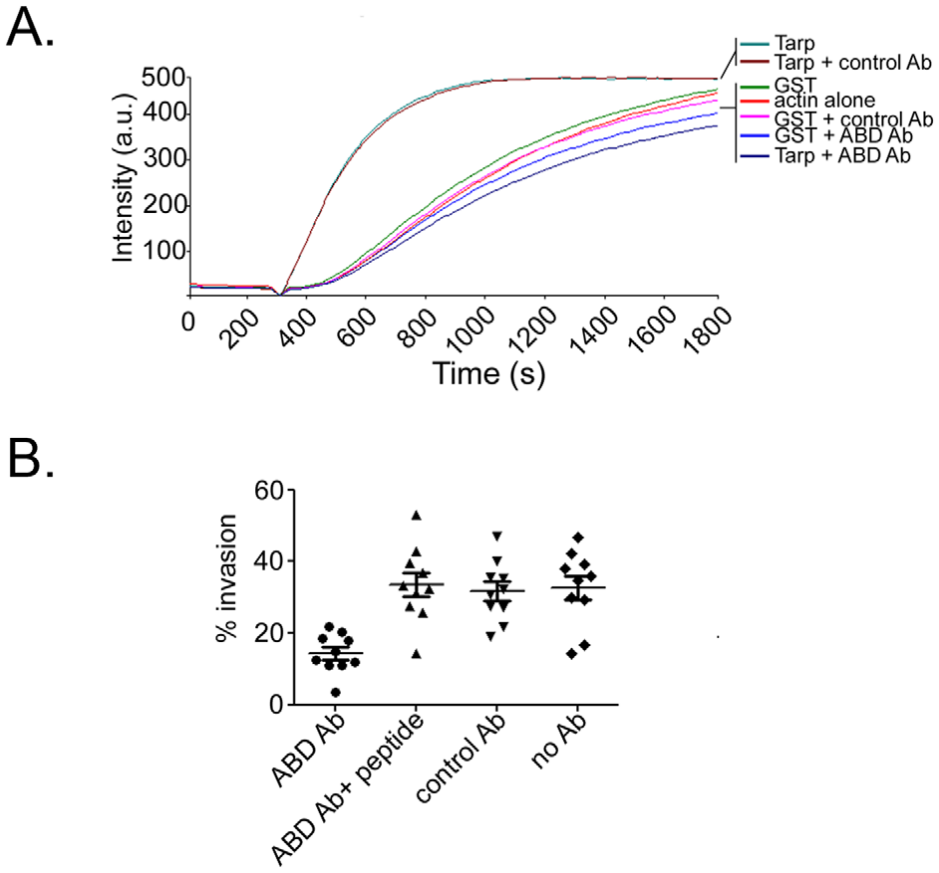
The Tarp actin binding domain (ABD) peptide antibody inhibits Tarp mediated actin polymerization *in vitro* and inhibits chlamydial entry *in vivo*. **A**) Tarp mediated actin nucleation (Tarp) was inhibited by the addition of Tarp actin binding domain specific antisera (Tarp+ABD Ab). Purified Tarp, GST and antibodies were added to 1µM pyrene conjugated actin and actin polymerization was measured as arbitrary fluorescence intensity (Intensity a.u.) over time (Time seconds) following the addition of polymerization buffer at 300 seconds. An irrelevant antibody did not alter Tarp (Tarp+control Ab) or GST (GST+control Ab) mediated actin polymerization. GST (GST) and actin alone (actin alone) served as additional controls. **B**) Graphical representation of EB invasion of ABD antibody pre-loaded HeLa cells. HeLa cells were pre-loaded with ABD or nonspecific control antibodies (control Ab) using a cationic lipid mixture (Pro-Ject Protein Transfection Reagent) to deliver the antibodies to the host cytosol. Intrinsically fluorescent CMPTX labeled EBs were used in invasion assays. After allowing for 30 min invasion, extracellular EBs were counterstained by indirect immunofluorescence with a monoclonal antibody to *C. trachomatis* L2 MOMP and a goat anti mouse antibody conjugated to Alexa 488. The percent of EB invasion (% invasion) was determined for cells harboring purified ABD (ABD Ab), ABD preincubated with an excess of the peptide immunogen (ABD Ab+peptide) and irrelevant control antibody (control Ab). Additional controls included untreated host cells (No Ab). The results are from one experiment representative of three separate experiments.

To investigate whether the ABD antiserum would disrupt the actin nucleating capabilities of Tarp, pyrene conjugated actin was used to examine the kinetics of actin polymerization as previously described [14]. Pre-incubation of purified L2 Tarp with ABD antiserum completely inhibited Tarp mediated actin polymerization (Figure 2A). These data indicate that the ABD antiserum is capable of neutralizing Tarp mediated actin polymerization *in vitro*.

The actin nucleating activity of the chlamydial Tarp effector within the host cell is presumed to play an important role in initiating actin polymerization at the site of EB attachment [9], [14]. To define a role for the Tarp actin binding domain and subsequent actin nucleation *in vivo*, affinity purified ABD antiserum was delivered into the cytosol of host cells as described in the Materials and Methods and the rate of EB invasion was determined. Host cells pre-loaded with the ABD antibody were significantly (P<0.0001) more resistant to EB invasion compared to host cells containing control antisera or ABD serum neutralized with the peptide immunogen (Figure 2B). These data suggest that the actin nucleating activity of Tarp is required for *C. trachomatis* invasion.

### The Tarp actin binding domain is a conserved feature among Tarp orthologs

**Figure 3:**
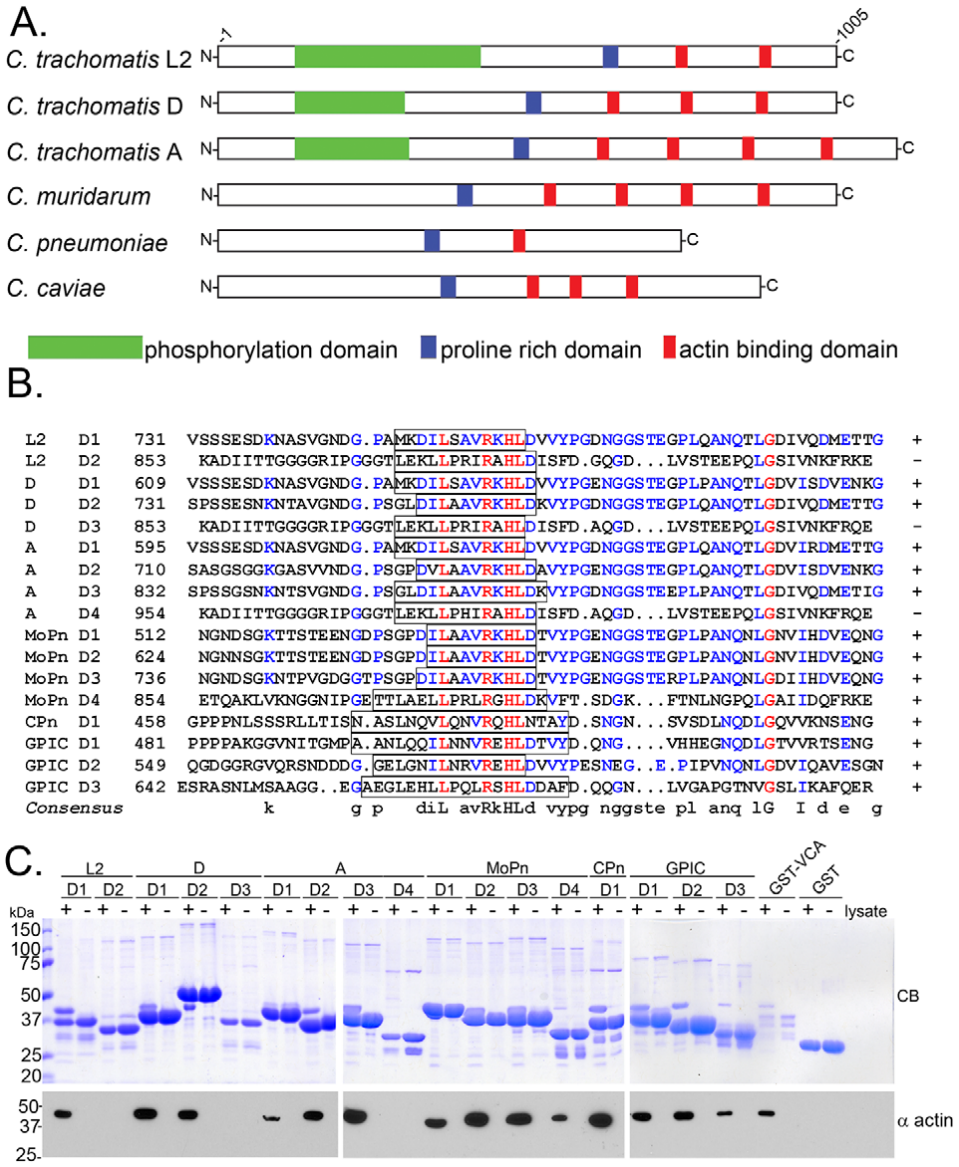
Tarp orthologs harbor multiple actin binding domains. **A**) A schematic of the Tarp orthologs from *C. trachomatis* serovar L2 (L2), *C. trachomatis* serovar D (D), *C. trachomatis* serovar A (A), *C. muridarum* (MoPn), *C. pneumoniae* (Cpn), and *C. caviae* (GPIC) indicating the location of the putative actin binding domains (red boxes), a proline rich domain (blue boxes), and tyrosine rich phosphorylation domain (green boxes). **B**) ClustalW sequence alignment of the putative actin binding domains from Tarp orthologs in A. The sequence predicted to harbor the actin binding alpha helix is indicated by the open box. Identical amino acids within each alignment are in red. Similar residues are in blue. The consensus sequence shown is based on homology greater than 50%. The number indicates the amino acid residue of the amino terminus of the peptide shown. **C**) The Tarp orthologs associate with actin. GST-fusions of the Tarp orthologs described above harboring sequence similar to the *C. trachomatis* L2 (L2) actin binding domain were expressed and purified. Extracts from HeLa cells were incubated with GST or GST fusions to Tarp orthologs and specifically bound proteins were resolved by SDS-PAGE and visualized by Coomassie blue staining (CB). Samples identical to those shown in the Coomassie-stained gel were subject to immunoblotting with an actin (α actin) specific antibody. A GST fusion to the VCA domain of N-wasp (GST-VCA) served as a positive control for actin binding.

All pathogenic chlamydial strains and serovars examined to date harbor a Tarp effector. Interestingly, the overall protein sequence of Tarp orthologs is quite divergent between chlamydial species [9], [16], [18]–[22]. However, the Tarp actin binding domain is present in all examined strains (Figure 3A). Sequence analysis indicates that each Tarp ortholog contains between 1 and 4 putative actin binding domains (Figure 3B). As previously reported, *C. trachomatis* L2 Tarp mediated actin nucleation is localized to a 200 amino acid domain encompassing the actin binding domain (ABD) and the upstream proline-rich domain (PRD) [14]. In order to determine if the putative actin binding domains present in the Tarp orthologs are functional, GST-Tarp fusions were generated for each of the predicted ABDs from *C. caviae*, *C. pneumoniae*, *C. trachomatis* serovar L2 *C. trachomatis* serovar D, *C. trachomatis* serovar A, and *C. muridarum*. Tarp actin binding domains were expressed individually, purified, and tested for their ability to precipitate host cell actin from soluble HeLa extracts (Figure 3C). All chlamydial Tarps contain at least one functional actin binding domain as determined by *in vitro* pulldown assays. Interestingly, in those *C. trachomatis* strains possessing multiple predicted ABDs, the most C-terminal predicted ABD in those Tarp orthologs was least similar in sequence to the functional ABDs and failed to precipitate actin in pull-down assays. *C. trachomatis* serovars D and A, *C. muridarum* and *C. caviae* harbor multiple functional actin binding domains whereas *C. trachomatis* L2 and *C. pneumoniae* harbor only a single functional ABD. These data demonstrate that all chlamydial Tarps are capable of binding actin and have the potential to influence host actin kinetics following translocation into host cells.

### Tarp orthologs demonstrate enhanced actin polymerization kinetics

**Figure 4:**
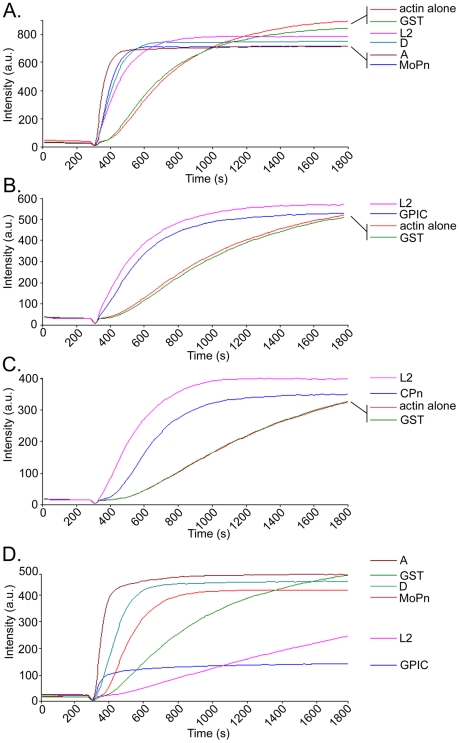
*C. caviae*, *C. pneumoniae* and *C. trachomatis* Tarp orthologs promote actin polymerization. **A**) Pyrene actin polymerization in the presence of GST-Tarp fusions. GST-Tarp fusions representing the C-domain of *C. trachomatis* L2 (L2), *C. trachomatis* serovar D (D) *C. trachomatis* A HAR13 (A) and *C. muridarum* (MoPn) were incubated with 1µM pyrene conjugated actin and actin polymerization was measured as arbitrary fluorescence intensity (Intensity a.u.) over time (Time seconds) following the addition of polymerization buffer at 300 seconds. GST and actin alone served as negative controls. **B**) Pyrene actin polymerization assays as A but with GST-Tarp fusions of the C-domain of *C. caviae* (GPIC), *C. trachomatis* L2 (L2), and negative controls GST, and actin alone. **C**) Pyrene actin polymerization assays as A but with GST-Tarp fusions of *C. pneumoniae* (CPn), *C. trachomatis* L2 (L2), and negative controls GST, and actin alone. **D**) Pyrene actin polymerization assays as A but with GST-Tarp fusions of Tarp orthologs lacking the proline rich domain. Note that all Tarp orthologs with greater than one actin binding domain increase initial rates of actin polymerization over the GST control. As previously shown [14], *C. trachomatis* L2 Tarp lacking the proline rich domain sequesters actin monomers to depress actin nucleation rates below control levels. The results are from one experiment representative of three separate experiments.

To determine if the Tarp orthologs were capable of manipulating the rate of actin polymerization *in vitro*, pyrene actin polymerization assays were used [23]. Tarp orthologs harboring sequences similar to the minimal 200 amino acid polymerization domain of *C. trachomatis* L2 (i.e. containing the proline-rich and actin binding domains) were tested for enhanced actin polymerization compared to GST and actin alone controls. The *C. trachomatis* serovars D, A and MoPn Tarp orthologs increased the rate of actin polymerization to levels comparable to L2 (Figure 4A). Likewise, GST-Tarp fusions of *C. caviae* and *C. pneumoniae* increased the rate of actin polymerization compared to controls although not to the same degree as L2 (Figure 4B,C). These data demonstrate that all chlamydial Tarps examined promoted actin polymerization *in vitro* suggesting that all chlamydial species and serovars directly manipulate host cytoskeletal dynamics. Actin nucleation requires alignment of actin monomers to initiate the formation of an actin filament. The 200 amino acid L2 Tarp fragment required for actin nucleation contains only a single actin binding domain and therefore necessitates oligomerization of the L2 Tarp peptide to nucleate actin. The proline rich domain is believed to be responsible for L2 Tarp oligomerization [14]. To determine if the Tarp orthologs which contain multiple actin binding sites are capable of actin nucleation in the absence of the proline-rich domain, GST-Tarp fusions were generated lacking the proline-rich domain and tested for their ability to nucleate actin. All Tarp orthologs which contain multiple actin binding sites were able to nucleate actin despite removal of their respective proline-rich domains (Figure 4D). These data suggest that while all Tarp orthologs are capable of actin nucleation, they may employ unique mechanisms of actin nucleation.

### Oligomerization of Tarp with multiple ABDs is not required for actin nucleation

**Figure 5:**
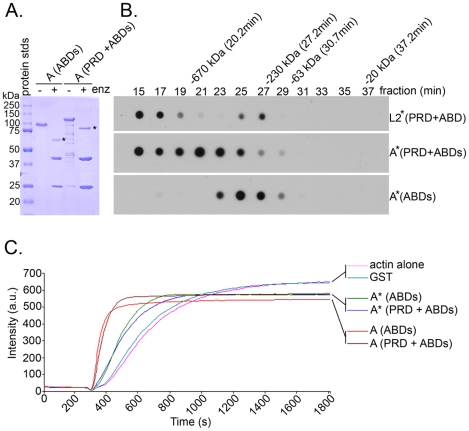
The *C. trachomatis* serovar A Tarp ortholog employs a spire-like actin nucleation mechanism and does not require the L2 Tarp proline rich domain for actin nucleation. *C. trachomatis* serovar A Tarp fragments harboring either the three functional actin binding domains (ABDs) alone or the actin binding domains and the proline rich domain (PRD) were digested to remove the GST moiety and analyzed by gel filtration and pyrene actin polymerization assays. **A**) *C. trachomatis* serovar A GST-Tarp fusion proteins were purified and digested with protease (+/− enz) to remove the GST moiety (* indicates GST is removed). Proteins were resolved by SDS/PAGE and visualized by Coomassie blue staining. **B**) Removal of the proline rich domain from *C. trachomatis* A Tarp inhibits oligomerization. Gel filtration of proteins shown in panel **A**. Protein fractions were collected in 2-min intervals from gel filtration columns and immobilized to a nitrocellulose membrane by vacuum filtration. Membranes were subjected to immunoblotting with a Tarp specific antibody. Protein standards are indicated above the dot-blot with respective molecular weight and peak elution times. **C**) Oligomerization of *C. trachomatis* A Tarp is not required for actin nucleation. Purified Tarp (A) with and without proline rich domain increased actin polymerization compared to GST and actin alone controls in pyrene actin polymerization assays. The results are from one experiment representative of three separate experiments.

*C. trachomatis* L2 Tarp, which contains only a single actin binding site, must oligomerize to catalyze actin nucleation [14]. Because the Spire protein of *Drosophila*, which contains four actin binding WH2 domains on a single polypeptide, has been reported to function independently as an actin nucleator [24], we examined the ability of Tarp fragments from *C. trachomatis* serovar A, whose Tarp protein harbors three functional ABDs, to nucleate actin filament formation in *in vitro* pyrene-actin fluorescence assays (Figure 5). Serovar A Tarp fragments bearing only the three ABDs or the three ABDs plus the proline-rich oligomerization domain were expressed as GST fusions, purified, and the GST domain cleaved from the Tarp (Figure 5A). Serovar A Tarp fragments bearing only the ABDs or the PRD plus the ABDs were applied to gel filtration sizing columns and fractions collected for analysis of Tarp fragment presence by dot immunoblot as previously described for serovar L2 Tarp domain analysis [14] (Figure 5B). Similar to L2 Tarp, serovar A Tarp fragments containing the proline-rich domain formed large oligomeric complexes while fragments containing only the ABDs did not (Figure 5B). However, in contrast to L2 Tarp fragments [14], both the fragments containing ABDs alone and the fragments containing the ABDs plus the PRD nucleated actin filament formation in pyrene-actin assays (Figure 5C). *C. trachomatis* A Tarp therefore appears to have the potential to utilize a hybrid mechanism involving actin nucleation by the alignment of at least three actin monomers on a linear polypeptide as well as actin polymerization by oligomerization of the Tarp protein due to the presence of the proline-rich oligomerization domain which is indispensible for those Tarp orthologs harboring only a single ABD.

## Discussion

Many bacterial pathogens modulate actin dynamics of eukaryotic host cells as a virulence mechanism. These pathogenic processes may involve manipulation of the cytoskeleton to promote internalization, inhibition of actin polymerization to prevent phagocytosis, or utilization of actin filamentation to promote intracellular movement and cell-to-cell spread of cytosolic pathogens. In most cases, bacteria that stimulate actin filament formation directly or indirectly do so through the recruitment of host actin nucleating machinery [25]. Recently, it has been recognized that certain bacterial proteins display actin nucleating activity independently of host factors [14], [15], [26]–[28]. The mechanism of chlamydial Tarp actin nucleation appears to be distinct from known eukaryotic or bacterial actin nucleators [14]. *C. trachomatis* L2 Tarp contains a single WH2 actin binding domain and requires an upstream proline-rich domain for oligomerization, which presumably brings multiple actin monomers into apposition to nucleate actin filament formation [14]. Surprisingly, Tarp of some chlamydial strains and species contains multiple actin binding domains on a single polypeptide thus may utilize a hybrid mechanism of actin nucleation involving binding of multiple actin monomers to a single polypeptide but potentially synergistic with oligomerization mediated by the upstream proline-rich domain. Notably, the actin binding function of Tarp appears to be essential to chlamydial pathogenesis as inhibition of its activity by cytosolic anti-ABD antibody is inhibitory to internalization of EBs.

Factors that nucleate actin filament formation do so through the stabilization of at least three actin monomers to overcome the kinetic barrier to nucleation. Eukaryotic cells possess three general classes of actin nucleation factors; the Arp2/3 complex, formins, and Spire [29], [30]. Each of these initiates actin filament formation by distinct mechanisms. The Arp2/3 complex nucleates branched filaments from existing actin filaments [31]. This is in contrast to Tarp, which nucleates linear filaments [14]. Formins nucleate formation of linear actin filaments and possess two characteristic formin homology domains, FH1 and FH2 [32], [33], which are not found in Tarp. The Spire protein of Drosophila also nucleates formation of linear actin filaments but does so through a mechanism involving four sequential WH2 domains on a single peptide [24]. More recently, it has been shown that Spire functions in close association with the Drosophila formin, Cappuccino [34]. As additional eukaryotic and bacterial proteins with actin nucleating activity have been discovered, it has become apparent that many of these novel nucleators do not fall clearly into of the above classes but may possess a combination of functional domains to create an actin nucleator employing hybrid mechanisms of actin nucleation [15]. For example, Cordon-Bleu, a vertebrate-specific nucleator important in neuronal development, contains three WH2 domains and six proline-rich domains [35]. Leiomodin-2, another recently recognized nucleator found in heart and skeletal muscle, is characterized by an N-terminal actin binding helix similar to that of tropomodulins, a leucine-rich repeat domain that binds actin, and a C-terminal WH2 domain [36]. Bacterial proteins with actin nucleating activity include *Salmonella* SipC [26], *Vibrio* VopF [28], and VopL [27], and chlamydial Tarp [14]. Although the mechanisms of actin nucleation differ, all but SipC share a common theme of the presence of one or more WASP Homology 2 (WH2) actin binding domains [15]. VopF and VopL each contain three WH2 domains and two proline-rich domains with the most N-terminal of these showing similarity to formin homology domain 1 (FH1) [27], [28]. Chlamydial Tarp shows no homology to formins but, as we demonstrate here, possesses a variable number of WH2 domains.

Comparison of the sequenced Tarp orthologs reveals a surprising diversity of isomers. *C. trachomatis* L2 Tarp contains six tyrosine-rich repeats while serovars D and A contain only three [9], [18],. *Chlamydia pneumoniae*, *C. caviae*, *C. muridarum* Tarp proteins lack the tyrosine-rich repeat domain and are not tyrosine phosphorylated [16]. Sequence analysis of all known Tarp orthologs indicates that the proline rich domain and actin binding alpha helix are conserved [16]. However, some Tarp orthologs harbor multiple actin binding domains. All Tarp orthologs examined harbored at least one actin binding domain that associated with actin derived from HeLa cell extracts. Furthermore, all Tarp orthologs increased initial rates of actin polymerization *in vitro* compared to actin alone and GST controls. Surprisingly, the *C. caviae* and *C. trachomatis* serovars A and D Tarp orthologs contained multiple functional actin binding domains suggesting a spire-like actin nucleation mechanism involving multiple WH2 domains on a single peptide may be employed by some Tarp orthologs. The presence of multiple ABDs could potentially allow one Tarp protein to align multiple actin monomers together to initiate an actin filament. While Tarp fragments harboring multiple actin binding domains nucleated actin *in vitro* even in the absence of the proline rich oligomerization domain, this domain appears to be conserved thus the proline-rich domain likely plays an essential role in promoting actin nucleation *in vivo*.. The biological advantage to strains bearing different numbers of Tarp WH2 domains is unclear. Indeed, the extent of variation within known *C. trachomatis* serovars or between clinical isolates is unknown. Intrinsic differences in Tarp structure are perhaps not surprising given that chlamydiae differ both clinically and biologically, demonstrating diverse tissue tropisms and varying degrees of localized and systemic infection [1], [37].

To confirm the role of the Tarp effector *in vivo*, an antibody was raised to a peptide overlapping the *C. trachomatis* L2 Tarp WH2 actin binding domain.. Antibody specific for the Tarp actin binding domain completely neutralized Tarp mediated actin nucleation *in vitro*. Furthermore, delivery of the ABD serum into host cells prior to a chlamydial infection significantly reduced the number of invasive bacteria. These data suggest that intracellular ABD antibody is capable of neutralizing the Tarp actin nucleating activity following translocation into the host but prior to bacterial entry. Defining essential virulence determinants in pathogens like chlamydiae that lack tractable genetic systems is often difficult. The inhibition of entry by intracellular antibody to a functional domain implies a role for Tarp as a virulence determinant of chlamydiae.

In addition to Tarp, chlamydial entry is also dependent on signal transduction cascades initiated following activation of host Rho family GTPases culminating in the activation of host cell Arp2/3 complex [38]–[41]. Because host cell Arp2/3 complex is also required for *C. trachomatis* invasion, we have proposed that the chlamydial and cellular actin nucleating activities function in concert to promote chlamydial invasion [14], [40]. Tarp nucleates the formation of linear actin filaments [14], whereas host Arp2/3 nucleates actin filaments that grow from the side of existing linear filaments, thus the two mechanisms may function synergistically. Although the role of phosphorylated Tarp in entry and the recruitment of Arp2/3 is somewhat controversial [16], [17], , Tarp proteins secreted from *C. pneumoniae* and *C. caviae* are not phosphorylated and therefore presumably either do not activate the Arp2/3 complex or activate Arp2/3 by an alternate signaling pathway. Furthermore, inhibition of *C. trachomatis* L2 Tarp tyrosine phosphorylation had no adverse effect on entry [17]. The signaling cascades initiated by the different chlamydiae appear to be species specific. *C. trachomatis* invasion requires the Rac GTPase while *C. caviae* requires both Rac and Cdc42 [39], [41]. Potentially related to the biodiversity exhibited by various chlamydial species, Tarp orthologs harbor domains that are species specific but variable in number such as the tyrosine rich repeat domain and conserved domains like the actin binding alpha helical domain that also varies in number [16]. Despite the observed differences in phosphorylation of the tyrosine rich repeat domain, the actin binding domain identified in all Tarp orthologs examined is conserved and a testament to the significance of this domain in chlamydial biology.

The Tarp effectors from various chlamydial species harbor distinct and conserved features such as the tyrosine rich repeat domain and the actin binding domain, respectively. In this study we demonstrate that all Tarp orthologs examined share the ability to bind and nucleate actin directly. This conserved feature is required for efficient invasion of host cells. Further elucidation of how Tarp and the Arp2/3 complex cooperate to produce a successful invasion is required to fully characterize the chlamydial entry mechanism. Careful analysis of the unique features of various Tarp orthologs may provide insight into differences observed within chlamydial species regarding tissue tropism and dissemination. Taken together our findings suggest that the type III secreted effector protein Tarp plays a vital role in chlamydial entry of human cells.

## Materials and Methods

### Organisms and cell culture

*Chlamydia trachomatis* serovar L2 (LGV 434), serovar D (UW-3-Cx), serovar A (HAR-13), *C. muridarum*, *C. caviae*, and *C. pneumoniae* (CWL029) were propagated in HeLa 229 cells and purified by Renografin density gradient centrifugation [45].

### Cloning, protein expression and purification

DNA fragments encoding the putative actin binding domain(s) of the Tarp orthologs from *C. pneumoniae* CWL029 (CPn0572) (Gly^440^-Val^540^), (Met^1^-Lys^755^), *C. caviae* (CCA00170) (Leu^460^-Asn^560^), (Lys^540^-Ser^640^), (Ser^640^-Pro^740^), (Thr^366^-Gln^666^), (Leu^460^-Pro^740^), *C. muridarum* (TC0741) (Glu^500^-Asp^600^), (Thr^620^-Asp^720^), (Asp^720^-Gly^820^), (Asn^840^-Glu^940^), (Ser^400^-Ala^827^), (Glu^500^-Glu^940^), *C. trachomatis* biovar *A* HAR-13 (CT456) (Asp^580^-Thr^680^), (Pro^700^-Ala^800^), (Ala^820^-Val^920^), (Thr^940^-Asp^1040^),(Ser^489^-Ser^926^),(Asp^580^-Val^920^); biovar D UW-3-Cx (CT456) (Ser^600^-Glu^700^), (Glu^700^-Gln^820^), (Gln^820^-Lys^940^), (Ser^503^-Ser^825^), (Ser^600^-Lys^940^); and biovar L2 LGV 434 (CT456) (Asp^726^-Ser^825^), (Pro^826^-Lys^940^), (Ser^625^-Ser^825^),(Thr^425^-Ser^825^), (Asp^726^-Lys^940^) were generated by PCR. In frame glutathione-S-transferase (GST) fusion proteins were generated by PCR amplifying the corresponding coding regions from *Chlamydiae* genomic DNA (Qiagen genomic purification kit, Valencia CA) using custom synthesized oligonucleotide primers (Integrated DNA Technologies, Coralville, IA) engineered with BamHI, EcoRI or XhoI linkers. PCR products were purified (Qiagen), digested with restriction enzymes (New England Biolabs, Beverly, MA) and subcloned into linearized pGEX-6P-1 to generate translational fusions with GST at the C-terminus.

pGEX-6P-1 plasmids encoding the Tarp orthologs were transformed into BL21 strain of *E. coli* (Novagen, Madison WI). Protein expression and purification were performed according to the procedures outlined in the Bulk GST Purification Module (GE Health Sciences, Piscataway, NY).

### GST fusion pull-down experiments

HeLa 229 cells were suspended in 100mM KCl, 10mM HEPES (pH 7.7), 2mM MgCl_2_ and 2mM ATP (buffer A) and disrupted by sonication delivered in four consecutive bursts of 20 second intervals on setting #4 (Ultrasonic Sonicator Processor XL equipped with a microtip: Misonix Incorporated, Farmingdale, NY). Insoluble material was removed by centrifugation (12,000 rcf, 25 min., 4°C). Glutathione-sepharose beads were incubated with 10µg of GST fusion proteins or GST for 1 hour at 4°C in PBS. (GE Health Sciences). GST-fusion protein coated beads were washed twice with PBS and once with buffer A prior to the addition of approximately 100µg of HeLa extract. Extracts and beads were incubated together for 2 hours at 4°C, washed three times with fresh buffer A and bound proteins were eluted using sample buffer.

### SDS-PAGE, immunoblotting and antibodies

Proteins were separated on SDS-10% polyacrylamide gels and transferred to 0.45µm pure nitrocellulose transfer and immobilization membrane (Schleicher & Schuell, Keene, NH). Immunoblotting employed peroxidase conjugated secondary antibodies (Chemicon International, Temecula, CA) and Supersignal West Pico chemiluminescent substrate (Pierce, Rockford, IL). The anti-actin C4 monoclonal antibody was purchased from Chemicon International. Polyclonal rabbit antibodies directed towards *C. trachomatis* L2 LGV 434 Tarp (CT456) was developed at Rocky Mountain Laboratories as previously described [9]. Rabbit antibodies directed toward the Tarp actin binding domain were generated against the peptide sequence GPAMKDILSAVRKHL and antigen-affinity purified by Sigma Genosys (Spring, TX).

### Immunoprecipitation

Fifty µl of mouse anti- WAVE2 (L-32) (Santa Cruz Biotechnology, Santa Cruz, CA) or mouse anti-Tarp (F07G2) was added to 50 µl of premixed protein A and G coated sepharose fast flow beads in 500 µl of PBS (GE Healthcare Bio-Sciences AB, Piscataway, NJ). Following a 1 hour incubation at 4°C, antibody coated beads were washed with fresh PBS. 1×10^7^ HeLa control cells and 1×10^7^ HeLa cells infected with *C. trachomatis* L2 were solubilized in RIPA buffer (150 mM NaCl, 1.0% IGEPAL CA-630, 0.5% sodium deoxycholate, 0.1% SDS, and 50 mM Tris, pH 8.0). Insoluble material was removed by microcentrifugation (15,000 rcf, 10 minutes, 4°C). Soluble lysate was added to the washed beads and incubated at 4°C for 3 hours. Beads were washed four times with RIPA buffer and the protein coated beads were suspended in 100 µl of protein sample buffer. Precipitated proteins were resolved on SDS PAGE and transferred to nitrocellulose membranes for immunoblotting with antibodies specific for the Tarp actin binding domain or WAVE2 (Chemicon, Temecula, CA)

### Dot blots

1×10^7^ HeLa control cells, 1×10^7^ HeLa cells infected with *C. trachomatis* L2, purified *C. trachomatis* L2 elementary bodies and recombinant Tarp protein were solubilized in RIPA buffer (150 mM NaCl, 1.0% IGEPAL CA-630, 0.5% sodium deoxycholate, 0.1% SDS, and 50 mM Tris, pH 8.0). Insoluble material was removed by microcentrifugation (15,000 rcf, 10 minutes, 4°C). Equal volume of soluble material was placed onto nitrocellulose membranes and immunoblotting was performed with antibodies specific for Tarp, the Tarp actin binding domain, WAVE2 or actin.

### Pyrene assay

The rate of actin polymerization in the presence of GST-fusions was monitored according to the methods outlined in the Actin Polymerization Biochem Kit from Cytoskeleton (Denver, CO). Briefly, monomeric pyrene labeled actin was prepared by diluting 100 µg of lyophilized pyrene actin into 2 mls of 5 mM Tris (pH 8.0), 0.2 mM CaCl_2_, and 0.2 mM ATP (G-buffer) and incubating for 1 hour at room temperature followed by an additional hour of incubation at 4°C. Monomeric pyrene actin was obtained by collecting the supernatant following a 2 hour, 100,000 rcf, 4°C spin in a Beckman Optima TLX Ultracentrifuge using a TLA 55 rotor (Beckman Coulter Inc., Fullerton, CA). Approximately 20 µg of pyrene labeled actin was gently mixed with 5 µg of GST fusion proteins in a volume of 500 µl for 10 minutes prior to the addition of 1/20^th^ volume of polymerization buffer (500 mM KCl, 20mM MgCl_2_, and 10mM ATP). The reaction was monitored over one hour with an LS 50B Luminescence Spectrophotometer directed by FL WinLab software version 4.0 (Perkin Elmer, Beaconsfield, BUCKS, UK) with 2.5 nm bandwidth at 365 nm excitation wavelength and 2.5 nm bandwidth at 407 nm emission wavelength.

### Peptide antibody delivery with Pro-Ject Protein Transfection Reagent

Pro-Ject Protein Transfection Reagent was used according to the manufactures instructions to deliver antibodies into host cells (Pierce, Rockford IL). Briefly, 1×10^5^ HeLa cells grown on glass coverslips were given 1µg of actin binding domain or *Rickettsia rickettsii* specific antibodies premixed with transfection reagent for 3 hours. Cascade blue conjugated dextran was added to the premixed transfection reagent to identify transfected cells by immunofluorescence. Additional controls included delivery of actin binding domain antisera neutralized with an excess of the peptide immunogen and PBS alone control.

### Invasion assay

*C. trachomatis* invasion of HeLa cells was determined essentially as previously described using intrinsically fluorescent, CMPTX-labeled *C. trachomatis* EBs [40]. Briefly, CellTracker (red) CMTPX labeled *C. trachomatis* L2 EBs (MOI ∼50) were permitted to attach to antibody-loaded target cells for 30 min at 4°C. The cultures were rinsed with cold HBSS and the temperature shifted to 37°C by the addition of pre-warmed RPMI plus 10% FBS. The cultures were then incubated at 37°C. The cultures were fixed with 4% paraformaldehyde at room temperature for 15 min and rinsed with PBS. The cells were not permeabilized. Extracellular EBs were labeled for 1 hour with a monoclonal antibody specific for chlamydial major outer membrane protein (MOMP). After four washes in PBS, secondary antibody conjugated to Alexa 488 was added for 1 hour. Coverslips were rinsed and mounted in ProLong Gold antifade reagent (Invitrogen, Carlsbad, CA). Cells were examined with a Nikon Microphot-FXA microscope equipped with phase contrast and epifluorescence optics. Images were obtained using a Photometrics CoolSnap HQ camera and processed using Adobe Photoshop CS2. Percentage internalized was taken as total EBs (red)−extracellular EBs (green)/total EBs (red)×100.

### Gel filtration

TARP peptides suspended in 1× PBS or buffer A were added to a Superdex 200 10/300 GL gel filtration column (Amersham Biosciences) controlled by a BioCAD Sprint Perfusion Chromatography System (PerSeptive Biosystems Inc., Framingham, MA) as previously described [14]. Eluted proteins were monitored by A280 absorbance and peak fractions harboring eluted Tarp were confirmed by immunoblot analysis.

### Accession numbers

Accession numbers for Tarp orthologs used here are as follows: *C. trachomatis* serovar L2, stain LGV434 - AAT47185, *C. trachomatis* serovar D, strain UW3/Cx - NP 219969.1; *C. trachomatis* serovar A, strain HAR-13 - YP328278.1; *C. muridarum* strain Nigg - NP 297115.1; *C. caviae* strain GPIC- NP 829043.1; *C. pneumoniae* strain CWL029 - NP 224768.1.
